# Intratumoural spatial distribution of S100B + folliculostellate cells is associated with proliferation and expression of FSH and ERα in gonadotroph tumours

**DOI:** 10.1186/s40478-022-01321-y

**Published:** 2022-02-09

**Authors:** Mirela Diana Ilie, Alexandre Vasiljevic, Marie Chanal, Nicolas Gadot, Laura Chinezu, Emmanuel Jouanneau, Ana Hennino, Gérald Raverot, Philippe Bertolino

**Affiliations:** 1 Inserm U1052, CNRS UMR5286, University Claude Bernard Lyon 1, Cancer Research Center of Lyon, Lyon, France; 2 Endocrinology Department, “C.I. Parhon” National Institute of Endocrinology, Bucharest, Romania; 3 Pathology Department, Reference Center for Rare Pituitary Diseases HYPO, “Groupement Hospitalier Est” Hospices Civils de Lyon, Bron, France; 4 Pathology Research Platform, Department of Translational Research and Innovation, Centre Leon Berard, Lyon, France; 5 Pathology Department, Targu Mures Emergency Hospital, Targu Mures, Romania; 6 Histology Department, Pharmacy, Science and Technology of Targu Mures, University of Medicine, Targu Mures, Romania; 7 Neurosurgery Department, Reference Center for Rare Pituitary Diseases HYPO, “Groupement Hospitalier Est” Hospices Civils de Lyon, Bron, France; 8 Endocrinology Department, Reference Center for Rare Pituitary Diseases HYPO, Groupement Hospitalier Est” Hospices Civils de Lyon, Bron, France

**Keywords:** Tumour heterogeneity, Folliculostellate cells, S100B + cells, Tumour microenvironment, Gonadotroph adenoma

## Abstract

Folliculostellate cells are S100B-expressing cells with numerous functions in the normal anterior pituitary. These cells have also been identified in pituitary neuroendocrine tumours (PitNETs), where their precise role remains elusive. Here, we aimed to build a refined cartography of S100B-expressing cells to characterise their interpatient and intratumoural spatial distribution, and to start identifying their potential functions in PitNETs. High-throughput histological analysis of S100B-stained tumour sections of 54 PitNETs revealed a significant decrease in S100B + cells in PitNETs compared to the normal anterior pituitary. A Ki67 index ≥ 3, a mitosis count > 2/10 per high power fields, and a proliferative status, were all associated with fewer S100B + cells in gonadotroph tumours. Gonadotroph tumours also showed interpatient and intratumoural heterogeneity in the spatial distribution of S100B + cells. The existence of an intratumoural heterogeneity was further confirmed by the incorporation to our spatial analysis of additional markers: Ki67, FSH, LH, ERα and SSTR2. The tumour areas with fewer S100B + cells displayed a higher percentage of Ki67 + cells, whereas strong positive correlations were observed between S100B + , FSH + , and ERα + cells. Such spatial associations suggest that S100B + folliculostellate cells could play a role in gonadotroph tumorigenesis, and may contribute to the maintenance of tumour cells in a low proliferating, FSH + /ERα + differentiated state. Albeit, further in-depth functional studies are required to decipher the mechanisms underlying these spatial associations and to potentially identify a therapeutic use.

**Supplementary Information:**

The online version contains supplementary material available at 10.1186/s40478-022-01321-y.

## Introduction

Pituitary neuroendocrine tumours (PitNETs) are the second most common adult intracranial tumours. They account for around 15% of intracranial neoplasms and represent a major health issue with a prevalence of 1 in 1000 people [1–3]. Their diagnosis and the prediction of their behaviour are challenging due to differences in their cell-of-origin within the anterior pituitary gland (APG) (gonadotroph, corticotroph, and cells of the Pit1 lineage, namely somatotroph, lactotroph, and thyrotroph cells) and their secretory capacity (functioning or non-functioning lesions). Though patients suffering from Pit1 and corticotroph tumours have benefited from the development of effective therapeutic options, gonadotroph tumours, which originate from follicle-stimulating hormone (FSH)- and luteinizing hormone (LH)-producing cells, and account for up to 35% of all PitNETs [4, 5], currently lack effective medical treatments [6]. In addition, gonadotroph tumours may present multiple recurrences with an unpredicted time-course following their surgical resection (relapse at 5 years being seen in 10–40% of patients) [7, 8]. Moreover, a small percentage of PitNETs displays an aggressive behaviour that remains difficult to predict [9, 10], characterised by resistance and/or multiple recurrences despite multimodal treatments [9, 11]. Therefore, the identification of factors associated with and/or leading to an aggressive phenotype, as well as the identification of new therapeutic targets and novel treatment options are of particular importance.

The tumour microenvironment (TME) that includes blood and lymph vessels, immune cells, cancer-associated fibroblasts, as well as extracellular matrix components, and soluble molecules [12], represents an appealing prognostic tool and a therapeutic target, and has been extensively described in numerous cancers [13]. However, the knowledge on the TME of PitNETs is still limited [14, 15]. Folliculostellate (FS) cells are non-endocrine APG-resident cells, identified based on their morphology and immunoreactivity to the S100B protein [16, 17]. FS cells account for ~ 5% of the normal APG [18, 19], where they have numerous roles, including a supportive role [20], the regulation of hormone secretion [18], phagocytic properties [21], and the production of numerous cytokines and growth factors such as interleukin 6 (IL-6), follistatin, basic fibroblast growth factor, transforming growth factor β, and vascular endothelial growth factor [22]. FS cells were also described in PitNETs [23–25], suggesting they might play a role in these tumours. However, their function on tumorigenesis-related processes and their usefulness as a biomarker or as a therapeutic target remain underexplored.

Here, we assessed the presence and distribution of S100B + cells within the TME of PitNETs in order to provide a refined cartography of S100B + cells within tumours and between patients. We developed a high-throughput pipeline of single-cell histological analysis based on whole tumour serial sections obtained from a series of 54 surgically-resected PitNETs. We uncover an interpatient and an intratumoural heterogenous distribution of S100B + cells in gonadotroph tumours. In addition, our findings highlight the spatial relationships between the presence of FS cells and the proliferative capacity, as well as FSH and oestrogen receptor alpha (ERα) immunoreactivity of gonadotroph tumour cells, suggesting potential roles of S100B + cells in gonadotroph tumorigenesis.

## Material and methods

### Tumour and normal APG samples

All pituitary tumours (n = 54) were collected by the same surgeon (E.J.), and subsequently diagnosed in the Pathology Department of *Groupement Hospitalier Est, Hospices Civils de Lyon*, Lyon, France by a single pathologist (A.V.). Normal APG samples (n = 4) used as controls were obtained from the Pathology Department of *Targu Mures Emergency Hospital*, Targu Mures, Romania. These were collected during autopsies performed at 6–20 h after death on adult patients who have died suddenly, without prior known endocrine dysfunction. Those patients did not have traumatic head injury, documented prolonged multiple organ failure or cerebral ischemia prior to death, and they did not have evident signs of cerebral autolysis or incidental sellar/parasellar lesions.

### Clinicopathological data

S100B immunostaining was prospectively incorporated in the histopathological and immunohistochemistry (IHC) diagnostic routines. Clinicopathological data were collected from the medical files of patients included. The IHC diagnosis of different PitNET subtypes was based on hormone immunoreactivity: FSH, LH, prolactin, growth hormone, thyroid-stimulating hormone, and adrenocorticotropic hormone. When no hormone expression was identified, IHC for transcription factors (steroidogenic factor 1 (SF1), pituitary specific transcription factor (Pit1), and T-box transcription factor TBX19 (T-pit)), completed the diagnosis [26, 27]. For statistical analysis, tumours of the Pit1 lineage were considered together. Tumours were classified as functioning or non-functioning based on FSH, LH, and sex hormone levels; prolactin levels; growth hormone levels during the glucose tolerance test and insulin-like growth factor 1; thyroid-stimulating hormone and free thyroxine levels; free urinary cortisol, the response to dexamethasone suppression tests, and adrenocorticotropic hormone levels. Tumours were classified as proliferative (i.e., “proliferative status”) when at least two of three criteria of proliferation, namely Ki67 index ≥ 3%, mitoses n > 2/10 high power fields (HPFs) (400 × magnification), and positive p53 (> 10 strongly positive nuclei/10 HPFs), were present [28]. Invasion was defined as cavernous and/or sphenoid sinus invasion detected on magnetic resonance imaging and/or during surgery; for the cavernous sinus invasion detected on magnetic resonance imaging, the revised Knosp classification was used [29]. The grading of tumours was based on the published five-tiered clinicopathological classification (1a, non-invasive and non-proliferative; 1b, non-invasive, but proliferative; 2a, invasive, but not proliferative; and 2b, invasive and proliferative; 3, metastatic tumour) [28].

### Immunohistological staining

For IHC, staining was conducted on 4 µm serial sections obtained from formalin-fixed paraffin-embedded (FFPE) tissues through a routine protocol performed on a BenchMark® ULTRA immunostainer (Ventana Medical Systems Inc, Roche Diagnostics, Tucson, Arizona, USA). Detection of positive staining was done using diaminobenzidine (ultraView Universal DAB Detection Kit, Ventana Medical Systems Inc, Tucson, Arizona, USA) followed by haematoxylin counterstaining (Bluing Reagent, Ventana Medical Systems Inc, Roche Diagnostics, Tucson, Arizona, USA). Primary antibodies for IHC were as follow: S100B (Agilent Dako, #Z0311), FSH (Beckman Coulter Immunotech, #0373), LH (Agilent Dako, #M3502), Ki67 (Agilent Dako, #M7240), ERα (clone SP1, Ventana Medical Systems Inc.), and somatostatin receptor type 2 (SSTR2) (Abcam, #ab134152). For immunofluorescence (IF), staining was performed using standard protocols. Briefly, 4 µm-thick FFPE sections were first deparaffinised at 55 °C for 30 min prior to 5 min of incubation in xylene. Tissue sections were subsequently rehydrated in decreasing concentrations of ethanol (100%, 95% and 70%). Heat-induced epitope retrieval was performed using a citrate-based unmasking solution at pH 6.0 (Vector Laboratories, California, USA, #H-3300) prior to a 1 h blocking step and an overnight incubation with S100B (Abcam, #ab52642) and FSH (Beckman Coulter Immunotech, #0373) primary antibodies. Secondary antibodies coupled to fluorochromes (anti-mouse-AlexaFluor488, cat#712-546-151, and anti-rabbit-Cy3, cat#711–166-152, Jackson ImmunoResearch, UK) were then incubated for 30 min before mounting the sections with a DAPI counterstaining solution (Vector Laboratories, California, USA, #H-1200).

### Slide scanning and image analysis

Stained whole sections were scanned at 20 × using a Pannoramic Scan (3D HISTECH Ltd., Budapest, Hungary) or a Zeiss Axio Scan.Z1 (Carl Zeiss Microscopy GmbH, Jena, Germany). Scanned images were visualised with CaseViewer software (3D HISTECH Ltd., Budapest, Hungary) and Zen2 software (blue edition). Image analysis, subsequent segmentation, and staining quantification, were performed with HALO® software v.2.3.2089.27 (Indica Labs, New Mexico, USA). Our pathologist (A.V.) manually annotated the areas corresponding to tumour tissue and to adjacent APG tissue (when present). The regions with staining artefacts were excluded prior to performing a single-cell segmentation with HALO® CytoNuclear module v1.6. Segmentation was then double-checked by eye for each sample to ensure that cells were correctly identified before quantifying the percentage of positive cells out of the total number of cells present within the area analysed. Finally, spatial plots that illustrate each stained cell as an individual point, were generated.

### Statistical analysis

Statistical analysis was performed in R commander/R package version 3.5.1 (R-project, Vienna, Austria) and GraphPad Prism 5 (GraphPad, California, USA). Mann–Whitney test, Kruskal–Wallis test followed by Dunn’s post-test, Wilcoxon matched paired test, Student's t-test with Welch’s correction, and Spearman’s correlation were applied as indicated. *p* < 0.05 (adjusted *p* < 0.05 in case of multiple comparisons) was considered significant. Graphs were generated with GraphPad Prism 5 (GraphPad, California, USA).

## Results

### High-throughput histological single-cell analysis based on whole-slide quantification reveals a loss of S100B + cells in PitNETs in comparison to normal APG

**Fig. 1:**
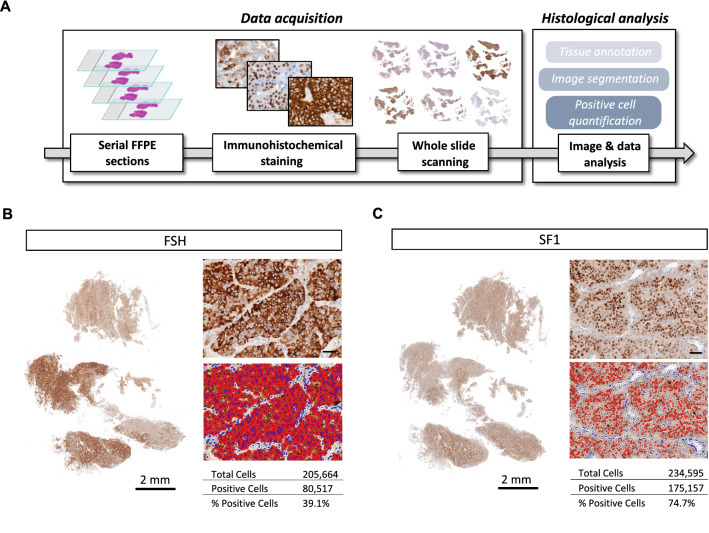
Schematic overview and representative analyses of our multi-marker histological quantification approach based on scanned whole-slides. **A** Working flow of our experimental/analysis pipeline. **B**, **C** Representative quantification of cytoplasmic (**B**) and nuclear (**C**) immunohistochemical staining using single-cell segmentation performed with the HALO® software (Indica Labs, New Mexico, USA) on scanned serial sections. Left panels show whole-section scans of immunohistochemical staining. Right panels are enlarged views of the immunohistochemical staining (top), with the matching image segmentation (bottom). Nuclei are segmented in blue, and positive staining is segmented in red. Quantification results are shown in the tables associated, as follows: total number of cells (counted on the total number of nuclei on the whole scan), number of positive cells, and the percentage of positive cells over the number of total cells. Scale bar of the enlarged images = 50 µm. Abbreviations: formalin-fixed paraffin-embedded (FFPE), follicle-stimulating hormone (FSH), steroidogenic factor 1 (SF1)

Quantification of immunoreactive cells on tissue sections is generally conducted either semi-quantitatively or by quantifying a limited number of selected fields. For the purpose of our study, we deemed such approaches to be imprecise, as the heterogeneous distribution of cells among tissues analysed can be overlooked. Thus, we developed a pipeline of IHC analysis to quantify single-cell staining on whole tumour sections (Fig. 1A). Analysis of FSH and SF1 expression of a gonadotroph tumour confirmed the feasibility of our technical approach, giving us the possibility to quantify and address the spatial distribution of immunoreactive cells in a large number (hundreds of thousands) of cells per section (Fig. 1B, C).

**Fig. 2:**
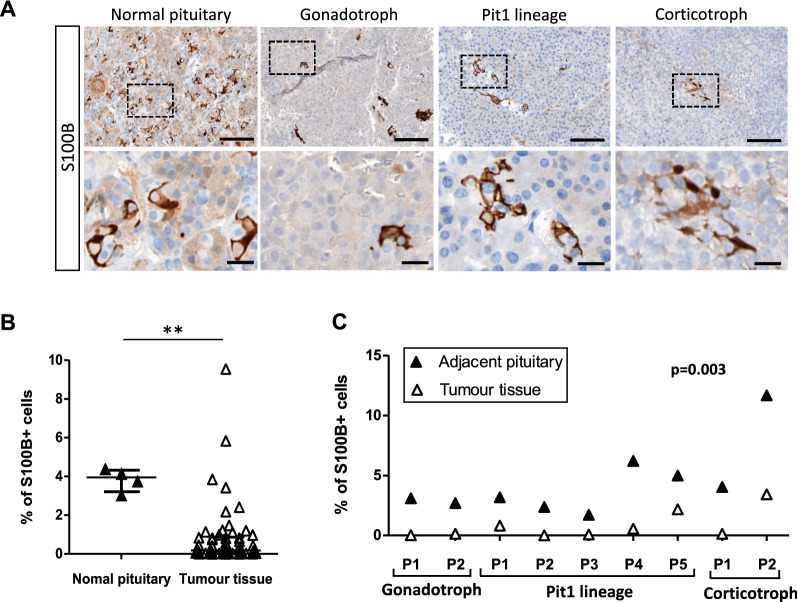
The percentage of S100B immunoreactive cells is lower in PitNETs compared to the APG. **A** Representative images of S100B immunoreactive cells found in the normal APG, and in gonadotroph, corticotroph, and Pit1 PitNETs. Lower panels show an enlarged view of the dashed insets. Scale bars = 20 µm (top), and 100 µm (bottom). **B** Percentage of S100B + cells in the tumour tissue of 54 PitNETs (median = 0.18%) versus 4 normal APG samples (median = 3.95%). Statistical test: Mann–Whitney, *p* = 0.003 (**). Graph—median with interquartile range. **C** Percentage of S100B + cells in matching tumour (median = 0.13%, range 0.00%-3.42%) versus adjacent APG tissue (median = 3.17%, range 1.71–11.68%) from 9 patients (P) with PitNETs. Statistical test: Wilcoxon matched paired test. Abbreviations: human pituitary neuroendocrine tumours (PitNETs), anterior pituitary gland (APG)

Following this validation, we analysed the number and the spatial distribution of FS cells through their S100B immunoreactivity, in a cohort of 54 patients with PitNETs (26 gonadotroph tumours, 20 tumours of the Pit1 lineage, and 8 corticotroph tumours) and 4 normal APG samples. The clinicopathological characteristics of the tumours analysed are detailed in Table 1. Quantification of S100B + cells on scanned whole-sections (Additional file 1: Fig. S1) confirmed that S100B + cells could be detected in normal APG, as well as in gonadotroph, corticotroph, and Pit1 tumours (Fig. 2A). Quantification of S100B + cells at the whole tumour level highlighted that tumour tissue showed a significant decrease in S100B + cells (median 0.18%) compared to normal APG (median 3.95%) (Fig. 2B). To refine this observation, we used histological sections in which both tumour and adjacent APG areas were present, and comparatively quantified the percentage of S100B + cells in individual patients. As shown in Fig. 2C, this quantification confirmed that the percentage of S100B + cells was lower in the tumour tissue compared to adjacent APG tissue, independently of the PitNET subtype. Interestingly, whereas most of the tumours analysed displayed a very low percentage of S100B + cells (43 tumours had < 1% of S100B + cells and 19 tumours < 0.1% S100B + cells), a small fraction of them had a higher percentage of S100B + cells reaching a maximum of 9.55% (Fig. 2B). Taken together, these observations suggest an interpatient heterogeneity regarding the percentage of intratumoral S100B + cells, which, nonetheless, is decreased in the majority of PitNETs in comparison to the normal APG.

**Table 1:** Clinicopathological characteristics of the tumours included in the study, considered together and by cell lineage

	All tumours n = 54	Gonadotroph n = 26	Pit1 lineage n = 20	Corticotroph n = 8
Age at surgery (years)	56 (27–87)	63 (37–87)	49 (27–77)	52 (33–66)
*Gender*				
Female	32 (59%)	12 (46%)	14 (70%)	6 (75%)
Male	22 (41%)	14 (54%)	6 (30%)	2 (25%)
*Sample localization*				
Tumour tissue	54 (100%)	26 (100%)	20 (100%)	8 (100%)
Adjacent anterior pituitary	9 (17%)	2 (8%)	5 (25%)	2 (25%)
Maximal diameter (mm)*	24 (5–60)	30 (20–55)	21 (7–60)	15 (5–32)
	n = 45	n = 22	n = 16	n = 7
*Invasion*				
Yes	28 (52%)	15 (58%)	9 (45%)	4 (50%)
No	18 (33%)	7 (27%)	9 (45%)	2 (25%)
NA	8 (15%)	4 (15%)	2 (10%)	2 (25%)
*Proliferation*				
No	46 (85%)	23 (88%)	16 (80%)	7 (88%)
Yes	8 (15%)	3 (12%)	4 (20%)	1 (12%)
*Ki67 index*				
< 3%	43 (80%)	22 (85%)	16 (80%)	5 (62%)
≥ 3%	11 (20%)	4 (15%)	4 (20%)	3 (38%)
*Mitosis*				
≤ 2/10 HPFs	47 (87%)	22 (85%)	18 (90%)	7 (88%)
> 2/10 HPFs	7 (13%)	4 (15%)	2 (10%)	1 (12%)
*p53*				
Negative	43 (80%)	24 (92%)	12 (60%)	7 (88%)
Positive	11 (20%)	2 (8%)	8 (40%)	1 (12%)
*Grade*				
1a	17 (31%)	6 (23%)	9 (45%)	2 (25%)
1b	1 (2%)	1 (4%)	0 (0%)	0 (0%)
2a	23 (43%)	13 (50%)	6 (30%)	4 (50%)
2b	5 (9%)	2 (8%)	3 (15%)	0 (0%)
NA	8 (15%)	4 (15%)	2 (10%)	2 (25%)
*Functioning status*				
Functioning	24 (44%)	0 (0%)	20 (100%)	4 (50%)
Non-functioning	30 (56%)	26 (100%)	0 (0%)	4 (50%)

^*^The only characteristic for which n is different than the one noted on the first row

The age at surgery and the maximal diameter are expressed as mean with range

Invasion = cavernous and/or sphenoid sinus invasion detected on magnetic resonance imaging and/or during surgery

Proliferation = two of three criteria of proliferation present, namely Ki67 index ≥ 3%, mitoses n > 2/10 high power fields (HPFs) (400 × magnification), and positive p53 (> 10 strongly positive nuclei/10 HPFs)

number (n), not available (NA)

### The presence of intratumoural S100B + cells is associated with lower proliferative properties and positively correlated with ERα expression in gonadotroph tumours

**Fig. 3:**
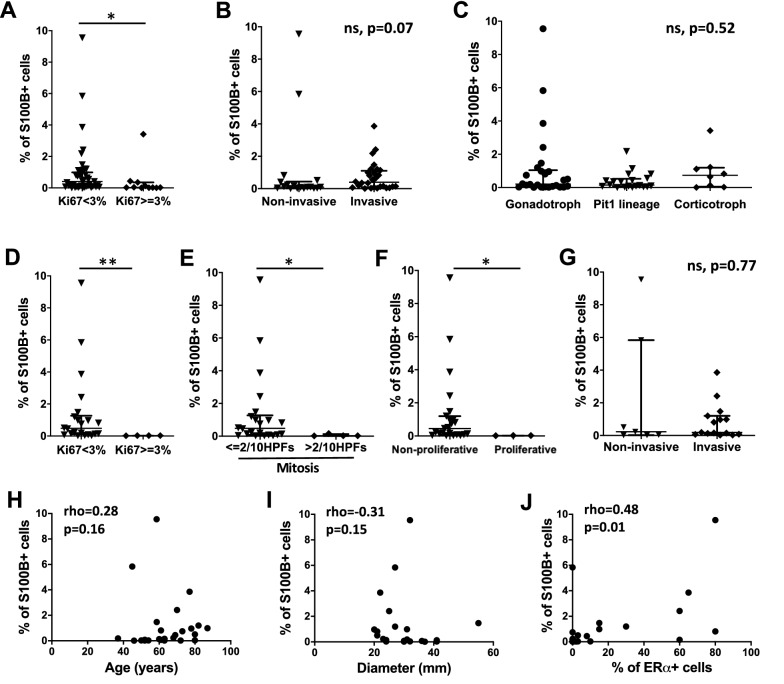
Association between clinicopathological characteristics and percentage of S100B + cells at the whole tumour level in PitNETs. **A** Percentage of S100B + cells in PitNETs with a Ki67 index < 3% (n = 43, median = 0.40%) versus a Ki67 index ≥ 3% (n = 11, median = 0.02%), *p* = 0.01 (*). **B** Percentage of S100B + cells in non-invasive (n = 18, median = 0.09%) versus invasive PitNETs (n = 28, median = 0.39%). **C** Percentage of S100B + cells in gonadotroph tumours (n = 26, median = 0.20%), tumours of the Pit1 lineage (n = 20, median = 0.14%), and corticotroph tumours (n = 8, median = 0.73%). **D** Percentage of S100B + in gonadotroph tumours with a Ki67 index < 3% (n = 22, median = 0.47%) versus a Ki67 index ≥ 3% (n = 4, median = 0.02%), *p* = 0.007 (**). **E** Percentage of S100B + cells in gonadotroph tumours with a number of mitosis ≤ 2/10 HPFs (n = 22, median = 0.47%) versus a number of mitosis > 2/10 HPFs (n = 4, median = 0.02%), *p* = 0.03 (*). **F** Percentage of S100B + cells in non-proliferative (n = 23, median = 0.44%) versus proliferative gonadotroph tumours (n = 3, median = 0.02%), *p* = 0.01 (*). **G** Percentage of S100B + cells in non-invasive (n = 7, median = 0.22%) versus invasive gonadotroph tumours (n = 15, median = 0.19%). **H** Correlation analysis between the percentage of S100B + cells and the age of patients in gonadotroph tumours (n = 26). **I** Correlation analysis between the percentage of S100B + cells and the maximal diameter in gonadotroph tumours (n = 22). **J** Correlation analysis between the percentage of S100B + cells and the percentage of ERα + cells in gonadotroph tumours (n = 25). Graphs: median with interquartile range (**A**–**G**), scatter plot (**H**–**J**). Statistical tests: Mann–Whitney (**A**–**G**), Kruskall-Wallis (**C**), Spearman’s correlation (**H**–**J**). Abbreviations: human pituitary neuroendocrine tumours (PitNETs), high power fields (HPFs), non-significant (ns)

We next investigated whether the percentage of intratumoural S100B + cells at the whole tumour level was associated with the clinicopathological characteristics of PitNETs analysed. We found that tumours with a Ki67 index ≥ 3% were associated with significantly fewer S100B + cells (*p* = 0.01) (Fig. 3A), whereas there was no association between the percentage of S100B + cells and the tumour invasive trait (Fig. 3B). We then wondered whether these observations were related to specific PitNET subtypes. Although the percentage of intratumoural S100B + cells was not statistically associated with the tumour subtype itself (Fig. 3C), a Ki67 index ≥ 3%, a mitosis count > 2/10 HPFs, and a proliferative status, were all significantly associated with a lower percentage of S100B + cells exclusively in gonadotroph tumours (Fig. 3D–F), but not in Pit1, nor in corticotroph tumours (Additional file 2: Fig. S2A-C and Fig. S2E-G). Moreover, S100B + cells were more widely spread in gonadotroph tumours (range 0.01–9.55%) compared to Pit1 (range 0–2.17%) and corticotroph tumours (range 0.007–3.42%). No association between invasiveness and the percentage of intratumoural S100B + cells was obtained when considering PitNET subtypes separately (Fig. 3G, Additional file 2: Fig. S2D and S2H). In addition, no statistical significance between the percentage of S100B + cells and other clinicopathological characteristics (age, gender, tumour dimension, grade, and the percentage of SSTR2 + or SSTR5 + cells) was reached in gonadotroph, corticotroph, or Pit1 tumours (Fig. 3H, I and data not shown); of note, SSTR5 was analysed only for corticotroph and Pit1 tumours. Interestingly, the percentage of S100B + cells showed a moderate positive correlation with the percentage of ERα + cells in gonadotroph tumours (Fig. 3J). These observations suggest that intratumoural S100B + cells may play a role in gonadotroph tumorigenesis-related processes.

### Spatial analysis of S100B + cell distribution highlights an intratumoural heterogeneity of S100B + cells

**Fig. 4:**
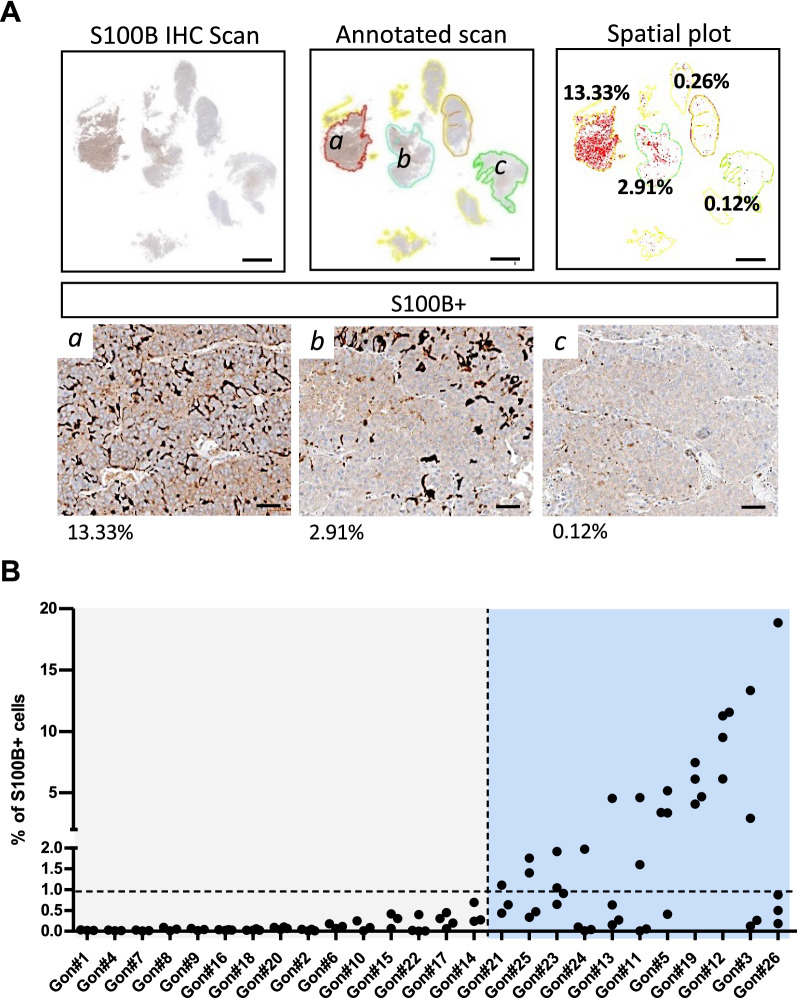
S100B + cells show an interpatient and an intratumoural heterogeneous distribution in gonadotroph tumours.** A** Quantification of S100B + cells on a gonadotroph tumour (Gon#3) showing a variation of more than 100-fold between different areas quantified. Top panel shows the four tumour areas (colour-coded in red, blue, orange, and green) that we defined and quantified on the scanned slide. The spatial plot represents each quantified cell as a red dot, and percentages for each quantified areas are noted. Lower panel shows enlarged views of S100B immunostaining in the indicated area (*a, b,* and *c*). Scale bars = 2 mm (top panel), 50 µm (lower panel). **B** Graph showing the percentage of S100B + cells quantified in multiple areas of 26 gonadotroph tumours (Gon#). Each dot plots the percentage of S100B + cells in a defined tumour area. Horizontal dashed line corresponds to a threshold of 1% S100B + cells. Vertical dashed line splits gonadotroph tumours with almost no expression of S100B among quantified areas and gonadotroph tumours presenting areas with variable percentages of detected S100B + cells. Abbreviation: immunohistochemistry (IHC)

Given that our results indicated a possible role for S100B + cells in gonadotroph tumorigenesis, we aimed at refining our cartography of this rare population of TME cells, by incorporating a spatial resolution to our analysis. In order to investigate the intratumoural distribution of S100B + cells in our cohort of 26 gonadotroph tumours, we defined for each sample multiple tumour areas on the same tumour slide. The percentage of S100B + cells was then quantified in each area, and the results subsequently confirmed by visually inspecting the stained areas (Fig. 4A). Analysis of the 26 gonadotroph tumours confirmed interpatient heterogeneity, with a subset of tumours displaying almost no S100B expression within the areas quantified and a group of lesions presenting areas with variable percentages of S100B + cells (Fig. 4A, B). Among the latter, the percentage of S100B + cells was heterogeneous between tumours, but also within the different areas of the same tumour, highlighting an intratumoural spatial heterogeneity of S100B + cells in gonadotroph PitNETs (Fig. 4).

### The intratumoural spatial distribution of S100B + cells is associated with FSH, ERα and Ki67 immunoreactivity

**Fig. 5:**
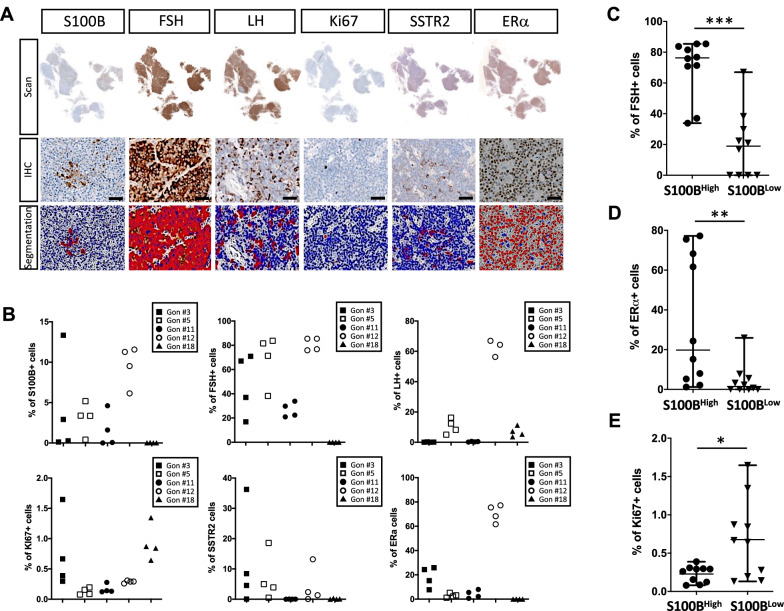
Spatial analysis of multiple markers provides insight into gonadotroph intratumoural heterogeneity and unravels spatial associations. **A** Representative images of scans (upper panel), enlarged views of IHC staining (middle panel), and resulting segmentations (lower panel), performed for S100B, FSH, LH, Ki67, SSTR2, and ERα, on serial tumour-sections. Scale bar = 50 µm. **B** For each of the five tumours (Gon#3, #5, #11, #12 and #18), we annotated the same tumour areas as previously done for the S100B marker (four areas per tumour). The graphs show the distribution of the S100B + , FSH + , LH + , Ki67 + SSTR2 + , and ERα + cells, in the resulting 20 independent tumour areas, revealing that these markers were either homogeneously, or heterogeneously distributed within each tumour. Each plotted symbol represents a tumour area. **C** Percentage of FSH + cells in S100B^High^ (median percentage of FSH + cells = 76.32%) versus S100B^Low^ areas (median percentage of FSH + cells = 18.93%), *p* = 0.0001 (***). **D** Percentage of ERα + cells in S100B^High^ (median percentage of ERα + cells = 19.78%) versus S100B^Low^ areas (median percentage of ERα + cells = 1.47%), *p* = 0.008 (**). **E** Percentage of Ki67 + cells in S100B^High^ (mean percentage of Ki67 + cells = 0.22%) versus S100B^Low^ areas (mean percentage of Ki67 + cells = 0.67%), *p* = 0.02 (*). S100B^High^ and S100B^Low^ expressing areas were separated by the median percentage of S100B + cells for the 20 areas, n = 10 for each group. Graphs are as follows: median with range (**C** and **D**), mean with range (**E**). Statistical tests: Mann–Whitney (**C** and **D**), Student’s t-test with Welch’s correction (**E**). Abbreviations: immunohistochemistry (IHC), follicle-stimulating hormone (FSH), luteinizing hormone (LH), somatostatin receptor type 2 (SSTR2), oestrogen receptor alpha (ERα)

The identification of a heterogeneous spatial distribution of S100B + cells in individual gonadotroph tumours led us to question whether the spatial distribution of S100B + cells may be of functional relevance in these tumours. To address this question, we built a cartography based on the IHC analysis of multiple markers on serial tumour sections, including five new markers, namely Ki67 (a proliferation marker), FSH and LH (the two hormones produced by gonadotroph cells), SSTR2 (a potential marker of response to treatment), and ERα (a transcription factor and a potential marker of response to treatment) [26, 30] (Fig. 5A). Spatial quantification of each marker was performed on 20 independent areas of 5 gonadotroph tumours detailed in Table 2 (4 areas per tumour). We chose the 5 gonadotroph tumours based on the percentages of S100B + cells: 4 tumours having a heterogeneous distribution, including the tumour having the widest spread of the percentages of S100B + cells—gonadotroph tumour #3, and one tumour having homogeneous percentages of S100B + cells—gonadotroph tumour #18.

**Table 2:** Clinicopathological characteristics of the 5 gonadotroph tumours for which serial tissue-sections were analysed

	Gonadotroph #3	Gonadotroph #5	Gonadotroph #11	Gonadotroph #12	Gonadotroph #18
Age at surgery (years)	77	87	58	58	52
Gender	Female	Male	Male	Male	Male
Maximal diameter (mm)	22	31	55	32	41
Invasion	Yes	Yes	Yes	No	Yes
Proliferation	No	No	No	No	Yes
Grade	2a	2a	2a	1a	2b

**Fig. 6:**
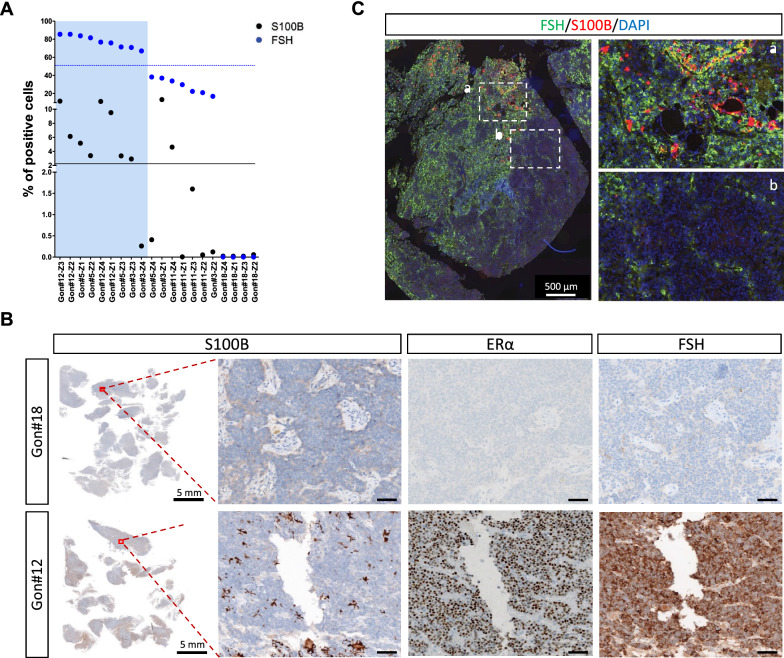
S100B-enriched areas are also enriched in FSH- and ERα-expressing cells in gonadotroph tumours. **A** Graph showing the percentage of S100B + cells and FSH + cells in 20 matching tumour areas (Z1 to Z4) of 5 gonadotroph tumours (Gon#3, #5, #11, #12 and #18). The areas represented on the right side (blue background) show the highest percentages of FSH + cells. Black and blue lines delineate the higher versus lower percentages of S100B + , and FSH + cells, respectively. The higher versus lower percentages of S100B + cells are split by the median. **B** Representative images of S100B, ERα, and FSH IHC staining performed on serial sections of two gonadotroph tumours. Left panel shows scanned S100B IHC slide. Right panels are enlarged views of the indicated areas (red squares). For each tumour, ERα and FSH staining are shown in the matching areas of serial sections. Scale bar = 50 µm. **C** Representative image of a double immunofluorescence staining for S100B (red) and FSH (green) performed on a gonadotroph tumour. Right panel shows enlarged views of the *a* and *b* dashed areas area. Abbreviations: immunohistochemistry (IHC), follicle-stimulating hormone (FSH), oestrogen receptor alpha (ERα)

The spatial quantification of each marker shown in Fig. 5B revealed their heterogeneous distribution between the different tumours. In these 5 tumours, the percentage of ERα-expressing and LH-expressing cells seemed to show less intratumoural heterogeneity than S100B-expressing, FSH-expressing, Ki67-expressing, and SSTR2-expressing cells (Fig. 5B). For each area, we further questioned the spatial association of the tested markers with the percentage of S100B + cells. Areas expressing low and high levels of S100B were defined based on the median percentage of S100B + cells within the 20 areas (n = 10 for each group). S100B^High^ areas had significantly more FSH + and ERα + cells (*p* = 0.0001, and *p* = 0.008, respectively) (Fig. 5C, D, Fig. 6A, Additional file 3: Fig. S3A) and fewer Ki67 + cells (*p* = 0.02) (Fig. 5E, Additional file 3: Fig. S3B). The percentage of LH + and SSTR2 + cells was not significantly different between S100B^High^ and S100B^Low^ tumour areas (Additional file 4: Fig. S4). Correlation analysis of the 20 tumour areas further confirmed that the percentage of S100B + cells was positively and strongly correlated with the percentage of FSH + cells (Spearman’s rho = 0.78, adjusted *p* = 0.0007) and of ERα + cells (Spearman’s rho = 0.76, adjusted *p* = 0.001) (Table 3). A strong positive correlation was also found between the percentage of FSH + cells and ERα + cells (Spearman’s rho = 0.67, adjusted *p* = 0.01) (Table 3), suggesting the existence of tumour areas simultaneously rich and poor in FSH + , ERα + , and S100B + cells (Additional file 3: Fig. S3C).

**Table 3:** Correlation matrix between the 6 markers analysed in 20 tumour areas of 5 gonadotroph tumours

	ERα%	FSH%	Ki67%	LH%	S100B%	SSTR2%
Spearman’s rho/adjusted *p*-value (Holm’s method)/n						
ERα%		**0.67/0.01/20**	− 0.10/1/20	0.01/1/19	**0.76/0.001/20**	0.40/0.72/20
FSH%	**0.67/0.01/20**		− 0.50/0.23/20	0.43/0.60/19	**0.78/0.0007/20**	0.55/0.12/20
Ki67%	− 0.10/1/20	− 0.50/0.23/20		− 0.21/1/19	− 0.31/1/20	0.01/1/20
LH%	0.01/1/19	0.43/0.60/19	− 0.21/1/19		0.31/1/19	0.13/1/19
S100B%	**0.76/0.002/20**	**0.78/0.0007/20**	− 0.31/1/20	0.31/1/19		0.34/1/20
SSTR2%	0.40/0.72/20	0.55/0.12/20	0.01/1/20	0.13/1/19	0.34/1/20	

The correlations with an adjusted p-value (Holm’s method) < 0.05 are written in bold letters

oestrogen receptor alpha (ERα), follicle-stimulating hormone (FSH), luteinizing hormone (LH), somatostatin receptor type 2 (SSTR2), number of patients for which the data are available (n)

To further validate these associations, we confirmed the existence of gonadotroph tumour areas presenting either an enrichment in S100B, ERα, and FSH immunoreactivity, or a lack of immunoreactivity for the three markers (Fig. 6B). Double IF S100B/FSH staining also revealed that S100B + cells were mainly located in areas enriched in FSH immunoreactivity (Fig. 6C). Hence, these observations confirmed the heterogeneous nature of gonadotroph tumours, and suggest that the heterogeneous distribution of S100B + cells might be of functional relevance within the TME of gonadotroph PitNETs.

## Discussion

The concepts of intratumoural, intertumoural and interpatient heterogeneity are intensively studied and acknowledged to be of tremendous importance in cancer biology [31, 32]. In PitNETs, tumour heterogeneity has only started to be unravelled by recent studies [30, 33, 34]. Here, our work contributes to extending this knowledge by providing an insight into the heterogeneity of PitNETs through the cartography of a rare TME component, namely S100B-expressing FS cells. Our work presents a novel high-throughput histological approach to analyse the spatial distribution and the association of multiple markers on scanned whole sections. To our knowledge, this report is the first to investigate the intratumoural spatial distribution of FS cells, and even gonadotroph intratumoural heterogeneity in general. As illustrated by our results, this kind of approach may refine our knowledge of tumour heterogeneity and favour the identification of functional associations between TME components and tumour cells.

Using whole-scan quantification of S100B IHC staining, we first confirmed an overall loss of S100B + FS cells in the majority of our PitNETs compared to normal APG and to adjacent APG, with 80% of these tumours having < 1% S100B + cells, and 35% having < 0.1% S100B + cells. This result is consistent with previous work that reported a loss of S100B + cells in PitNETs in comparison to normal APG collected from autopsies or from APG regions adjacent to tumour tissue [19, 35]. Interestingly, our analysis further revealed an interpatient heterogeneity with percentages of S100B + cells ranging from 0 to 9.55% in the 54 PitNETs analysed. Among PitNET subtypes, gonadotroph tumours had the most variable S100B distribution, though this may be partly due to our cohort composition.

Retrospective investigation of clinicopathological characteristics revealed that PitNETs with a Ki67 index ≥ 3% were associated with fewer S100B + cells. Further analysis comparing PitNET subtypes, pointed this proliferative trait to be driven by the gonadotroph subtype. Indeed, in gonadotroph tumours, not only was a Ki67 index ≥ 3% significantly associated with fewer S100B + cells, but also a mitosis count > 2/10 per HPFs, and the proliferative status. While we also found the overall percentage of S100B + cells to positively correlate with the percentage of ERα + cells in gonadotroph tumours, the overall percentage of S100B + cells was not associated with age, gender, invasion or tumour dimension in any of the PitNET subtypes, a result consistent with previous work by Voit et al. in acromegaly patients [25]. Moreover, the percentage of intratumoural S100B + cells was not statistically associated with the grade, and did not correlate with the percentage of SSTR2 + cells in any of the PitNET subtypes. In Pit1 and in corticotroph tumours, this percentage was not associated with proliferation markers, and did not correlate with the percentage of SSTR5 + cells, either. Of note, in gonadotroph tumours, it would have been interesting to also compare S100B + cells to SSTR3 + cells. Although the absence of statistically significant associations might be due to small sample sizes, these observations support nevertheless the hypothesis that S100B + cells may have distinct functions in different PitNET subtypes, and that the loss of these cells could favour the proliferative status of gonadotroph tumours.

Spatial analysis of cell distribution in different tumour areas of 26 gonadotroph PitNETs further revealed an intratumoural heterogeneity of S100B + cells. While we identified a first group of gonadotroph tumours with almost no S100B + cells, we found a second group that presented a wide distribution of S100B + cells ranging from 0.005 to 18.85% within different areas analysed. The presence of an intratumoural heterogeneity and the existence of low- and high-expressing S100B areas was intriguing and made us question the functional consequences of such a spatial cell distribution in gonadotroph tumours. To address this question, we added to our spatial analysis 5 new markers: Ki67, FSH, LH, ERα and SSTR2. Analysing 20 areas from 5 independent gonadotroph tumours, we found that higher percentages of S100B + cells were associated with lower percentages of Ki67. This observation was consistent with our previous observation based on the overall percentage of S100B + cells per tumour, and it further confirmed the functional relationship that may exist between gonadotroph tumour proliferation and the loss of S100B + cells.

More interestingly, through our spatial analysis, we observed strong positive correlations between the percentage of S100B + cells and FSH + cells, and between S100B + cells and ERα + cells, respectively. A similar correlation was also found between the percentage of FSH + cells and ERα + cells, suggesting the occurrence of tumour areas simultaneously rich and poor in FSH + , ERα + , and S100B + cells. Consistently, a positive correlation between FSH and ERα at the whole tumour level has already been reported in gonadotroph tumours [30, 36], suggesting that our technical approach is appropriate to investigate spatial associations of marker co-expression. Given the fact that ERα is also expressed in the vast majority of normal human gonadotroph cells which produce, secrete and stain for FSH [37, 38], we hypothesize that gonadotroph tumour cells expressing ERα and FSH might be closer to a normal phenotypic state than gonadotroph tumour cells lacking ERα and FSH expression. Identification of an enrichment in S100B + folliculostellate cells in areas also enriched in ERα-expressing and FSH-expressing cells may suggest a role for these cells in maintaining a more differentiated, functioning or closer to the normal phenotype state of gonadotroph tumour cells. This hypothesis appears to be coherent with our other findings, as the loss of intratumoural S100B + cells was associated with increased proliferation. Alternatively, FS cells may secrete cofactors that modulate the signalling of oestrogens through ERα [39] leading to FSH production, or FS cells may secrete molecules or physically interact with gonadotroph tumour cells leading to FSH production independently of ERα. Such molecules might be IL-6 or transforming growth factor β, both of which were shown to be produced by FS cells [22, 40], and to stimulate the secretion of FSH by the normal APG [40, 41]. Indeed, given that FS cells have been shown to modulate the hormone secretion of the normal APG [22] and that relationships between S100B + cells and the preoperative levels of growth hormone and of prolactin were revealed in acromegaly patients [25], it would not be surprising if FS cells also have an effect on hormone production/secretion by gonadotroph tumour cells.

Knowing that FS cells produce a large numbers of secreted cytokines, it would be tempting to speculate that FS cells could modulate tumour cell behaviour through paracrine signalling, by acting, for example, as a senescence inducer and/or proliferation controller. Interestingly, the secretion of the senescence-associated IL-6 cytokine by FS cells was shown to inhibit the proliferation of endocrine cells in the normal APG [40]. In pituitary tumours, it has been shown that IL-6 was mainly produced by tumour cells [40, 42], and that its silencing in primary cultures of human pituitary tumours decreased tumour senescence [43]. To the same extent, FS cells also produce follistatin [22], which may be another interesting candidate since dysregulation of follistatin function was shown to be linked to multiple cancers, including pituitary adenomas [44]. Our results highlight therefore the need for refining our knowledge on the secretome of intratumoural S100B + cells since it could have potent actions in PitNETs.

Aside from their possible function in gonadotroph tumours and other PitNETs, one may question whether FS cells may act as “gatekeeper” cells involved in maintaining normal structure and function of the APG, and whether their loss might favour the onset of pituitary tumours. Studies on the normal human APG have shown an increased presence of S100B + cells with age, especially after the age of 70 [45, 46], and proposed that the increased presence of these cells represents the successful modality of APG aging [45]. Given the multiple roles FS cells have in the normal APG through paracrine [17, 22] and physical actions [47, 48], it appears indeed possible that these cells are necessary to maintain a normal tissue structure and function. Their loss might therefore shift the balance from normal tissue preservation to adenoma formation/progression. And, reciprocally, a normal APG tissue might be required to maintain the presence of FS cells.

## Conclusions

We conducted an interpatient and an intratumoural cartography of S100B + cells to gain insight into the potential functions these cells might have in gonadotroph tumours. Concomitantly, we demonstrated the feasibility of analysing whole tumour sections at the single-cell resolution in human PitNETs, and of spatially analysing multiple markers on serial sections. The overall percentage of S100B + cells was associated with lower proliferative properties and positively correlated with ERα expression in gonadotroph tumours. However, the overall percentage of S100B + cells was not associated/correlated with age, gender, invasion, grade, tumour dimension and percentage of SSTR2 + cells in any of the PitNET subtypes. In Pit1 and in corticotroph tumours, this percentage was not associated with proliferation markers, and did not correlate with the percentage of SSTR5 + cells, either. Our work highlights a potential role for intratumoural S100B + cells in modulating the proliferation, the differentiation, and the hormonal production of gonadotroph tumour cells. It also suggests that S100B + cells may contribute to maintaining a phenotype closer to the normal tissue in certain tumour areas. However, the importance of S100B + cell loss in pituitary tumorigenesis remains to be demonstrated. Our work paves the way towards a refined view of gonadotroph tumour heterogeneity and towards a better understanding of the role of S100B + folliculostellate cells in gonadotroph tumours. It also emphasizes the need to perform high-resolution analysis of the TME at the single-cell level to unravel novel mechanisms that drive tumorigenesis-associated processes, which will hopefully lead to the discovery of new diagnostic and prognostic markers and of new and/or personalized treatments.

## Supplementary Information

**Additional file 1**: Fig. S1. Representative S100B immunohistochemical (IHC) staining and segmentation. **A**, Representative immunostaining patterns of S100B in tumour tissue and adjacent anterior pituitary. Left panel shows a scanned S100B immunostained section of a gonadotroph tumour. Right panels are magnified views of the observed staining in the adjacent anterior pituitary and tumour tissue present on the same slide (adjacent anterior pituitary is annotated in blue). Scale bar = 50 µm. **B**, Representative example of a single-cell segmentation performed with the HALO® software (Indica Labs, New Mexico, USA): S100B IHC staining (top), and the resulting segmentation (bottom) are shown. Nuclei are segmented in blue, while the positive staining is segmented in red.

**Additional file 2**: Fig. S2. Association between the percentage of S100B+ cells and clinicopathological traits in Pit1 and corticotroph tumours. **A**, Percentage of S100B+ cells in Pit1 tumours with a Ki67 index < 3% (n = 16, median = 0.14%) versus a Ki67 index ≥ 3% (n = 4, median = 0.19%). **B**, Percentage of S100B+ cells in Pit1 tumours with a number of mitosis ≤ 2/10 HPFs (n = 18, median = 0.11%) versus a number of mitosis > 2/10 HPFs (n = 2, median = 0.39%). **C**, Percentage of S100B+ cells in non-proliferative (n = 16, median = 0.14%) versus proliferative Pit1 tumours (n = 4, median = 0.19%). **D**, Percentage of S100B+ cells in non-invasive (n = 9, median = 0.09%) versus invasive Pit1 tumours (n = 9, median = 0.35%). **E**, Percentage of S100B+ cells in corticotroph tumours with a Ki67 index < 3% (n = 5, median = 0.83%) versus a Ki67 index ≥ 3% (n = 3, median = 0.13%). **F**, Percentages of S100B+ cells in corticotroph tumours with a number of mitosis ≤ 2/10 HPFs (n = 7, median = 0.64%) versus a number of mitosis > 2/10 HPFs (n = 1, median = 1.11%). **G**, Percentage of S100B+ cells in non-proliferative (n = 7, median = 0.64%) versus proliferative corticotroph tumours (n = 1, median = 1.11%). **H**, Percentage of S100B+ cells in non-invasive (n = 2, median = 0.07%) versus invasive corticotroph tumours (n = 4, median = 0.74%). Graphs show median with interquartile range. Statistical test: Mann-Whitney. Abbreviations: non-significant (ns), high power fields (HPFs).

**Additional file 3**: Fig. S3. Spatial association between S100B+, ERα+, Ki67+, and FSH+ cells. **A**, Graph showing the percentage of S100B+ cells and ERα+ cells in 20 matching tumour areas (Z1 to Z4) of 5 gonadotroph tumours (Gon#3, #5, #11, #12 and #18). The areas represented on the right side (red background) show the highest percentage of ERα+ cells. Black and red lines delineate the higher versus lower percentages of S100B+ cells, and ER + cells, respectively. The higher versus lower percentages of S100B+ cells are separated by the median. **B**, Graph showing the percentage of S100B+ cells and Ki67+ cells in 20 matching tumour areas (Z1 to Z4) of 5 gonadotroph tumours (Gon#3, #5, #11, #12 and #18). The areas represented on the right side (green background) show the highest percentages of Ki67+ cells. Black and green lines delineate the higher versus lower percentages of S100B+ cells, and Ki67+ cells, respectively. The higher versus lower percentages of S100B+ cells are separated by the median. **C**, Graph showing the percentage of S100B+ cells, FSH+ cells, and ERα+ cells, in 20 matching tumour areas (Z1 to Z4) of 5 gonadotroph tumours (Gon#3, #5, #11, #12 and #18). The 10 areas represented on the right side (grey background) show the highest percentage of S100B+ cells (the higher versus lower percentages of S100B+ cells are split by the median). Blue and red lines delineate the higher versus lower percentages of FSH+, and ERα+ cells, respectively. Abbreviations: follicle-stimulating hormone (FSH), oestrogen receptor alpha (ERα)

**Additional file 4**: Fig. S4. The percentage of LH+ and SSTR2+ cells was not associated with the percentage of S100B+ cells in gonadotroph tumours. **A**, Percentage of LH+ cells in S100B^High^ (n = 9, median percentage of LH+ cells = 12.32%) versus S100B^Low^ areas (n = 10, median percentage of LH+ cells = 2.01%). **B**, Percentage of SSTR2+ cells in S100B^High^ (n = 10, median percentage of SSTR2+ cells = 3.13%) versus S100B^Low^ areas (n = 10, median percentage of SSTR2+ cells = 0.08%). S100B^High^ and S100B^Low^ expressing areas were separated by the median percentage of S100B+ cells for the 20 areas. Graphs show median with range. Statistical test: Mann-Whitney, ns=non-significant. Abbreviations: luteinizing hormone (LH), somatostatin receptor type 2 (SSTR2).

## Data Availability

Not applicable.

## Abbreviations

APG: Anterior pituitary gland
FSH: Follicle-stimulating hormone
FS: Folliculostellate
FFPE: Formalin-fixed paraffin embedded
HPFs: High power fields
IF: Immunofluorescence
IHC: Immunohistochemistry
IL-6: Interleukin 6
LH: Luteinizing hormone
ERα: Oestrogen receptor alpha
PitNETs: Pituitary neuroendocrine tumours
SSTR2: Somatostatin receptor type 2
SF1: Steroidogenic factor 1
TME: Tumour microenvironment
