# Supplementary figures and images for: Intratumoural spatial distribution of S100B + folliculostellate cells is associated with proliferation and expression of FSH and ERα in gonadotroph tumours
Source: Acta Neuropathol Commun. 2022 Feb 9;10:18. doi: 10.1186/s40478-022-01321-y (PMC8827287; doi:10.1186/s40478-022-01321-y)

A

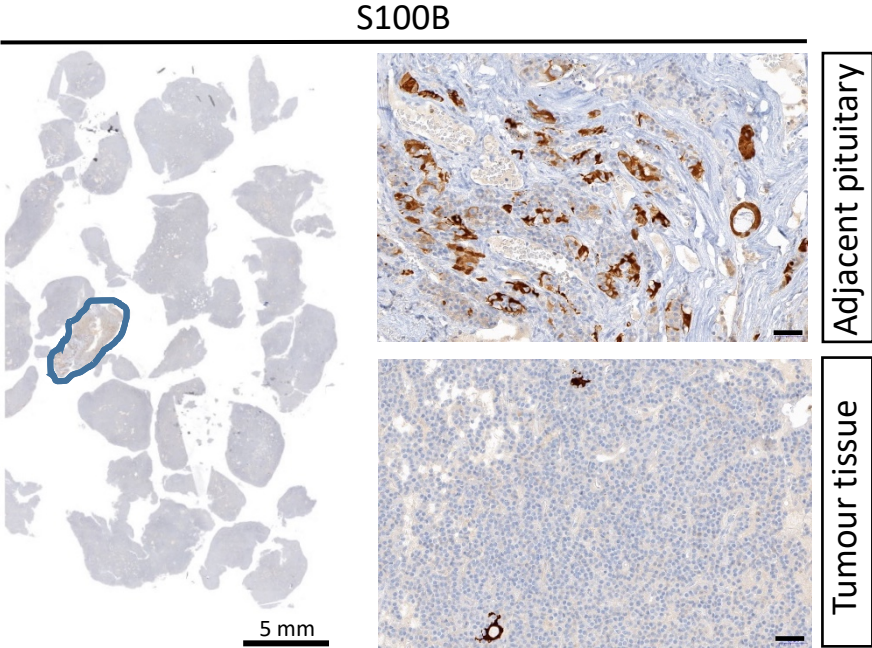

B

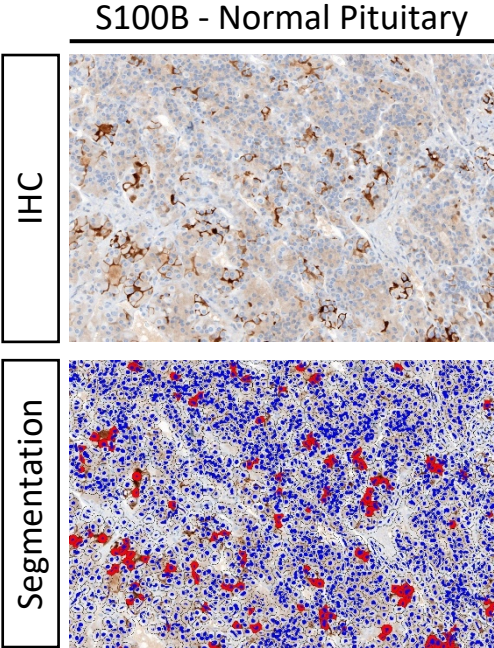

Supplement: Supplementary file 1 — Additional file 1: Fig. S1. Representative S100B immunohistochemical (IHC) staining and segmentation. A, Representative immunostaining patterns of S100B in tumour tissue and adjacent anterior pituitary. Left panel shows a scanned S100B immunostained section of a gonadotroph tumour. Right panels are magnified views of the observed staining in the adjacent anterior pituitary and tumour tissue present on the same slide (adjacent anterior pituitary is annotated in blue). Scale bar = 50 µm. B, Representative example of a single-cell segmentation performed with the HALO® software (Indica Labs, New Mexico, USA): S100B IHC staining (top), and the resulting segmentation (bottom) are shown. Nuclei are segmented in blue, while the positive staining is segmented in red. [file 40478_2022_1321_MOESM1_ESM.pdf]

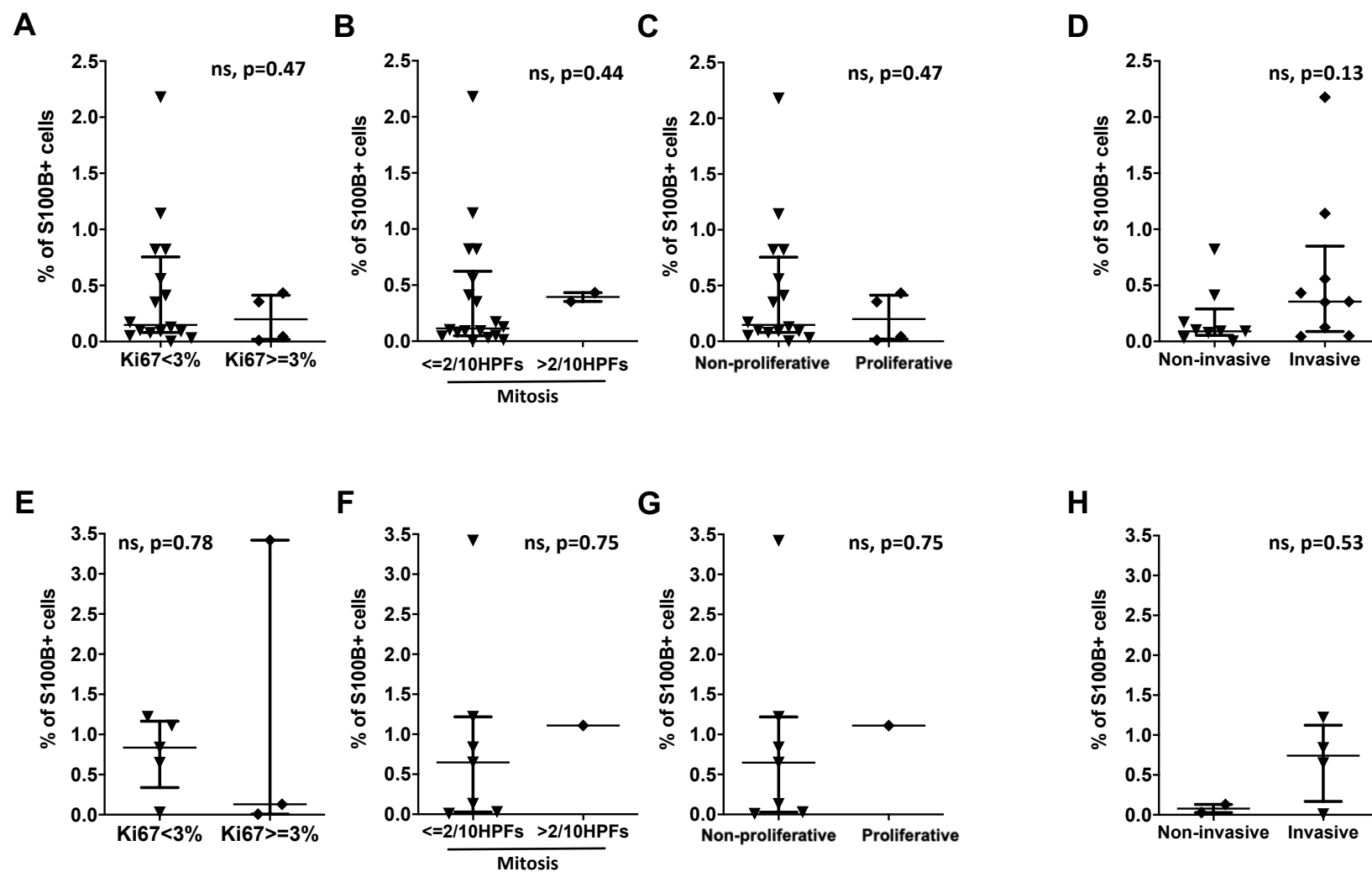

Supplement: Supplementary file 2 — Additional file 2: Fig. S2. Association between the percentage of S100B+ cells and clinicopathological traits in Pit1 and corticotroph tumours. A, Percentage of S100B+ cells in Pit1 tumours with a Ki67 index < 3% (n = 16, median = 0.14%) versus a Ki67 index ≥ 3% (n = 4, median = 0.19%). B, Percentage of S100B+ cells in Pit1 tumours with a number of mitosis ≤ 2/10 HPFs (n = 18, median = 0.11%) versus a number of mitosis > 2/10 HPFs (n = 2, median = 0.39%). C, Percentage of S100B+ cells in non-proliferative (n = 16, median = 0.14%) versus proliferative Pit1 tumours (n = 4, median = 0.19%). D, Percentage of S100B+ cells in non-invasive (n = 9, median = 0.09%) versus invasive Pit1 tumours (n = 9, median = 0.35%). E, Percentage of S100B+ cells in corticotroph tumours with a Ki67 index < 3% (n = 5, median = 0.83%) versus a Ki67 index ≥ 3% (n = 3, median = 0.13%). F, Percentages of S100B+ cells in corticotroph tumours with a number of mitosis ≤ 2/10 HPFs (n = 7, median = 0.64%) versus a number of mitosis > 2/10 HPFs (n = 1, median = 1.11%). G, Percentage of S100B+ cells in non-proliferative (n = 7, median = 0.64%) versus proliferative corticotroph tumours (n = 1, median = 1.11%). H, Percentage of S100B+ cells in non-invasive (n = 2, median = 0.07%) versus invasive corticotroph tumours (n = 4, median = 0.74%). Graphs show median with interquartile range. Statistical test: Mann-Whitney. Abbreviations: non-significant (ns), high power fields (HPFs). [file 40478_2022_1321_MOESM2_ESM.pdf]

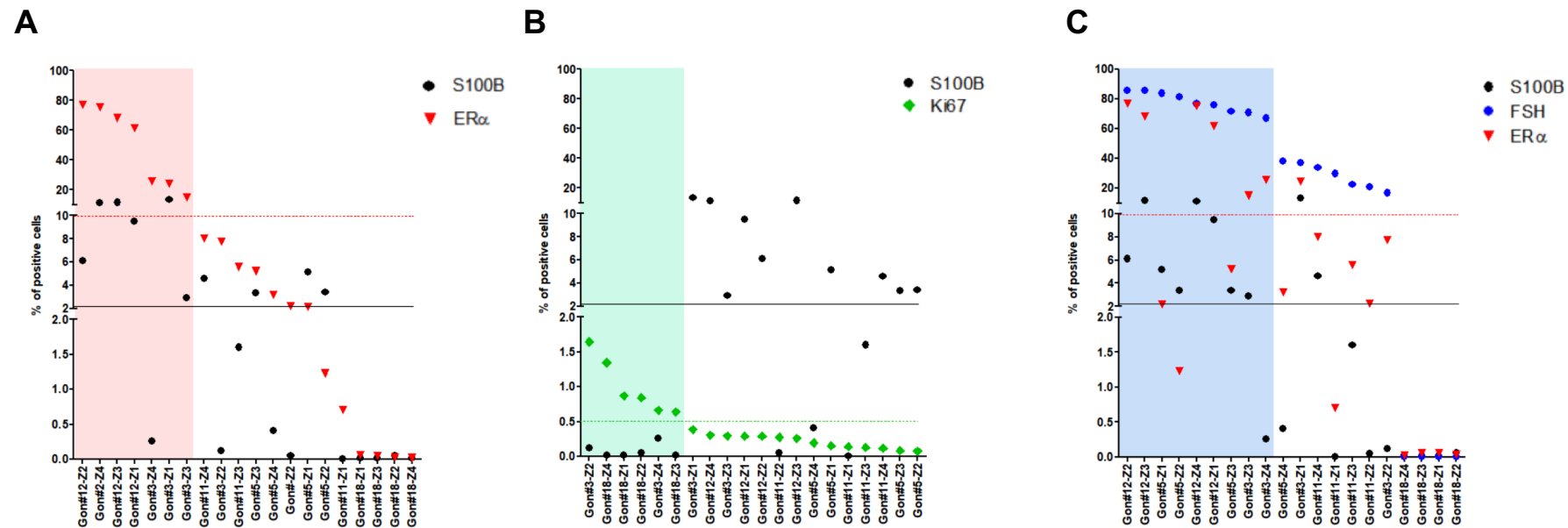

Supplement: Supplementary file 3 — Additional file 3: Fig. S3. Spatial association between S100B+, ERα+, Ki67+, and FSH+ cells. A, Graph showing the percentage of S100B+ cells and ERα+ cells in 20 matching tumour areas (Z1 to Z4) of 5 gonadotroph tumours (Gon#3, #5, #11, #12 and #18). The areas represented on the right side (red background) show the highest percentage of ERα+ cells. Black and red lines delineate the higher versus lower percentages of S100B+ cells, and ER + cells, respectively. The higher versus lower percentages of S100B+ cells are separated by the median. B, Graph showing the percentage of S100B+ cells and Ki67+ cells in 20 matching tumour areas (Z1 to Z4) of 5 gonadotroph tumours (Gon#3, #5, #11, #12 and #18). The areas represented on the right side (green background) show the highest percentages of Ki67+ cells. Black and green lines delineate the higher versus lower percentages of S100B+ cells, and Ki67+ cells, respectively. The higher versus lower percentages of S100B+ cells are separated by the median. C, Graph showing the percentage of S100B+ cells, FSH+ cells, and ERα+ cells, in 20 matching tumour areas (Z1 to Z4) of 5 gonadotroph tumours (Gon#3, #5, #11, #12 and #18). The 10 areas represented on the right side (grey background) show the highest percentage of S100B+ cells (the higher versus lower percentages of S100B+ cells are split by the median). Blue and red lines delineate the higher versus lower percentages of FSH+, and ERα+ cells, respectively. Abbreviations: follicle-stimulating hormone (FSH), oestrogen receptor alpha (ERα) [file 40478_2022_1321_MOESM3_ESM.pdf]

**A**

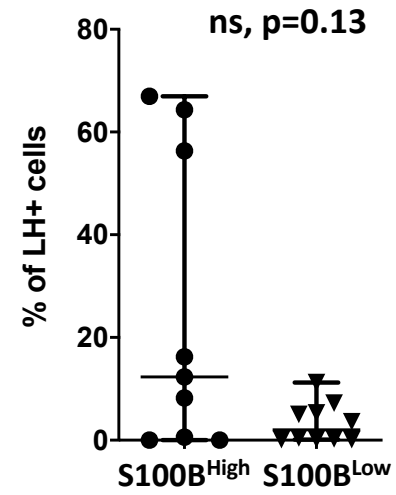

**B**

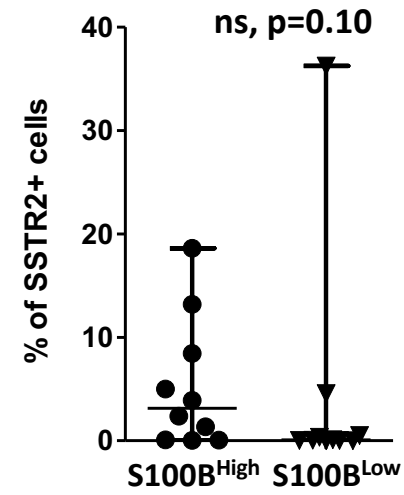

Supplement: Supplementary file 4 — Additional file 4: Fig. S4. The percentage of LH+ and SSTR2+ cells was not associated with the percentage of S100B+ cells in gonadotroph tumours. A, Percentage of LH+ cells in S100BHigh (n = 9, median percentage of LH+ cells = 12.32%) versus S100BLow areas (n = 10, median percentage of LH+ cells = 2.01%). B, Percentage of SSTR2+ cells in S100BHigh (n = 10, median percentage of SSTR2+ cells = 3.13%) versus S100BLow areas (n = 10, median percentage of SSTR2+ cells = 0.08%). S100BHigh and S100BLow expressing areas were separated by the median percentage of S100B+ cells for the 20 areas. Graphs show median with range. Statistical test: Mann-Whitney, ns=non-significant. Abbreviations: luteinizing hormone (LH), somatostatin receptor type 2 (SSTR2). [file 40478_2022_1321_MOESM4_ESM.pdf]
